# Transition from Military Service: Mental Health and Well-being Among Service Members and Veterans with Service-connected Disabilities

**DOI:** 10.1007/s11414-021-09778-w

**Published:** 2022-01-26

**Authors:** Gary R. Bond, Monirah Al-Abdulmunem, Robert E. Drake, Lori L. Davis, Thomas Meyer, Daniel M. Gade, B. Christopher Frueh, Ross B. Dickman, Daniel R. Ressler

**Affiliations:** 1 Westat, Rivermill Commercial Center, 85 Mechanic Street, Lebanon, NH 03766 USA; 2 Research Service, Tuscaloosa VA Medical Center, Tuscaloosa, AL USA; 3 Department of Psychiatry, University of Alabama School of Medicine, Birmingham, AL USA; 4 Sloan School of Management, Massachusetts Institute of Technology, Cambridge, USA; 5 Department of Public Administration and Policy, American University, Washington, DC USA; 6 Department of Psychology, University of Hawaii, Hilo, HI USA; 7 Department of Neurosurgery, Houston Methodist Academic Institute, Houston, TX USA; 8 Hire Heroes USA, Alpharetta, GA USA

**Keywords:** Veterans, Disability, Mental health, Depression, Transition

## Abstract

Transitioning from military service is stressful for veterans with service-connected disabilities seeking civilian employment. This descriptive study examined self-assessed mental health, well-being, and substance use of men and women shortly before or after transition from US military service, compared to norms from community and military samples. As part of a prospective study evaluating an innovative employment program, researchers interviewed 229 current and former service members with service-connected disabilities transitioning from U.S. military service. Compared to published norms, respondents reported significantly poorer outcomes on 5 of 6 standardized measures, indicating less life satisfaction, poorer mental health, more symptoms of depression and posttraumatic stress disorder, and greater financial distress. In the previous year, 42% were prescribed opioid medications, over twice the annual opioid prescription rate of 19% in the general US population. Systematic strategies are needed to ensure access for transitioning veterans with serious behavioral health issues to appropriate evidence-based practices.

## Introduction

Each year, approximately 200,000 men and women separate from the US military ^1^—a transition that many experience as challenging, especially post 9/11 veterans (i.e., active military service after September 2001). ^2,3^ In some surveys, over 60% of post-9/11 veterans have reported difficulty adjusting to civilian life, compared to 25% of veterans from earlier eras. ^4,5^ Transitioning veterans contend with dramatic changes to daily schedules, home life, and income, as well as the shift from military to civilian culture. ^6^ Another common worry of transitioning service members is financial security. Nearly one-third of post-9/11 veterans reported financial trouble in the last year, nearly twice the rate for pre-9/11 veterans. ^4,7–9^

Among the stressors experienced by transitioning service members are the challenges of finding employment. Upon discharge, many veterans are surprised and demoralized when they are rejected on scores of job applications, often extended over a period of months. Unemployment among veterans is associated with poorer mental health and well-being. The direction of causality is likely bidirectional, with extended periods of unemployment resulting in depression, anxiety, and other psychiatric symptoms, ^10,11^ while discouragement and despair undermine the motivation to seek employment. ^12^

In addition to these social, employment, and financial concerns, a growing number of veterans develop significant mental and physical health conditions during and after their service. ^13–16^ A 2016 survey of over 9000 newly separated veterans found that 53% reported chronic physical conditions and 33% reported chronic mental health conditions, with chronic pain, sleep problems, anxiety, and depression the most commonly endorsed problems. ^17^ Consistent with the opioid epidemic in the general society, veteran rates of opioid overdose deaths also have been increasing. ^18^ Given these complex challenges, many veterans may need outside help to manage the return to civilian life.

Veteran applications for and receipt of service-connected disability compensation from the Department of Veterans Affairs (VA) have been increasing dramatically among those discharged from active duty during the post-9/11 era. ^15^ In 2019, 4.7 million veterans received compensation from the Veterans Benefits Administration for a service-connected disability, including 1.8 million (41%) of all post 9/11 veterans, more than double the rate for older veterans. ^19^ The severity of disability ratings has also been increasing. ^20^ The percentage of veterans with a cumulative disability rating of 60% or higher nearly doubled from 2013 to 2019 (30% vs. 55%). ^19,21^

Despite a large literature on VA health services, research on the transition period from military to civilian life is surprisingly limited. Moreover, research has not yet adequately documented the scope of behavioral health and physical health problems among transitioning veterans or identified who is at most risk for behavioral health problems. A recent review found few studies comparing veterans to civilian populations on mental and physical well-being. ^16^ The purpose of the current study was to identify the prevalence and severity of behavioral health symptoms in a self-selected sample of service members transitioning from the military with service-connected disabilities and wanting help finding suitable employment. The current study compared self-reported health and well-being in the study sample to published norms on widely used standardized measures from available surveys of civilian and veteran samples.

## Methods

### Overview

The current study was a secondary analysis of baseline data collected for a prospective national evaluation of an employment program for veterans. Outcomes based on follow-up interviews will be reported in subsequent papers. The Westat Institutional Review Board approved the study, which followed the principles outlined in the Declaration of Helsinki.

### Recruitment and enrollment

The study included an opportunity sample of enlisted men and women transitioning from the military recruited to participate in an evaluation of an innovative employment program. Eligibility criteria included under the age of 45, at least 6 months of active military service with an honorable or general discharge, within 6 months before separation or 12 months after separation, and receiving or applying for a veterans disability compensation disability rating. At study enrollment, participants were either transitioning service members without civilian employment or veterans who were unemployed or working in temporary jobs.

Trained interviewers conducted all research interviews by telephone. Enrollment procedures included email contact initiated by prospective participants, a screening interview, informed consent, and study enrollment, followed by the baseline interview.

Interviewers identified potential participants through letters, social media, online sources, and word of mouth. Using mailing lists from two data repositories maintained by the VA, recruitment letters were sent to 28,000 recently discharged veterans. Online advertisements directed prospective respondents to a study website featuring a self-administered, qualifying survey (a series of screening questions that helped determine eligibility) and an invitation to those passing the screening questions to send contact information to the research team.

### Background characteristics

The research team obtained detailed information on demographics and military service, adapting questions from prior studies. ^22^ When first interviewed, participants were in various stages of applying for and receiving VA disability ratings; thus, interviewers obtained initial disability ratings over the course of the baseline and subsequent interviews.

### Self-report measures

#### Satisfaction with life scale (SWLS)

This 5-item scale is a widely used self-report scale to measure life satisfaction. ^23^ The SWLS has good convergent and discriminant validity and temporal stability. ^24^ The internal consistency coefficient (Cronbach’s alpha) for SWLS in the study sample was .85.

#### Veterans Rand-12 (VR-12)

The VR-12 is a 12-item self-reported assessment of health widely used in veteran populations. ^25^ It is a slight modification of the well-validated SF-12. ^26,27^ The VR-12 includes two subscales providing a general self-assessment of mental health status (mental component score [MCS]) and physical health status (physical component score [PCS]). MCS and PCS scores are based on statistically derived algorithms. ^25^ The internal consistency coefficients (Cronbach’s alpha) for MCS and PCS were .89 and .85, respectively, in the study sample.

#### Patient health questionnaire-9 (PHQ-9)

The PHQ-9 is a 9-item self-report depression checklist that has been well validated in two large studies and has been used in many medical surveys. ^28^ It is also used in routine clinical practice as a screener for depression. According to the scale’s developers, a score of 10 or more indicates moderate depression. The internal consistency coefficient (Cronbach’s alpha) for PHQ-9 was .88 in the study sample.

#### Incharge financial distress financial well-being (IFDFW)

This 8-item checklist measures financial distress/financial security. ^29^ The checklist has good psychometric properties, including content and construct validity and sensitivity to change. ^30^ The internal consistency coefficient (Cronbach’s alpha) for IFDFW was .95 in the study sample.

#### PTSD checklist for DSM-5 (PCL-5) with criterion A

The PCL-5 is a 20-item checklist used to screen for a DSM-5 diagnosis of posttraumatic stress disorder (PTSD) as defined by the Diagnostic and Statistical Manual, Version 5. ^31^ Its psychometric properties have been found to be satisfactory. ^32^ The standard cut-off score on the PCL-5 indicating probable PTSD is greater than or equal to 33. ^33^ In the current study, participants could opt out of completing the PCL-5, resulting in a reduced sample size. The internal consistency coefficient (Cronbach’s alpha) for PCL-5 was .63 in the study sample.

#### Substance use

Respondents were asked questions regarding use *in the last year* of the following substances: tobacco, alcohol, marijuana and cannabis products, illegal drugs, and prescribed opioids. The interview also included questions about *any use* of the identified substances, *frequency of use* in the last week for the first four substances, and the *duration of use* for prescription opioids.

### Norms for outcome measures

The study design did not include a matched comparison group to serve as case controls for the study sample. In lieu of a case control design, the research team searched the published literature for norms to use as comparators for the self-report measures of health and well-being used in this study. Several scales are used widely to screen for symptoms signaling the need for treatment; the percentage of the study sample exceeding the cut-off scores are reported for these scales. When available, the analyses used norms based on military or veteran samples. Some published reports also provided norms for subgroups, which were used to match the study sample on one or more background characteristics, specifically age and veteran status. Available published data did not adequately control for a range of other possible confounds (e.g., time since separation from the military, sex, or education). The norms used in this report were drawn from the following published studies:

- *Satisfaction with life scale (SWLS).* A survey of a national representative sample of 5399 adults found that SWLS ratings varied little by sex, age, race/ethnicity, or education. ^34^ SWLS ratings are lower, however, in samples of people in psychiatric treatment. ^24^ The norm used in the current study was the mean SWLS score of 28.0 found in a survey of 136 veterans and service members transitioning from the military. ^35^
- *Veterans Rand-12 (VR-12).* To develop norms for the VR-12, researchers constructed a large, well-defined and nationally representative sample of the US population (*N* = 173,221) participating in three national surveys. ^25^ The current study used the norms for this sample (50.1 for MCS and 39.8 for PCS). ^25^
- *Patient health questionnaire-9 (PHQ-9).* A study examined the prevalence of depression, as measured by the PHQ-9 in a sample of 1885 US veterans participating in a national survey. ^36^ For the current analysis, the comparison group was the subgroup of 304 veterans in the youngest age group of 25 to 44 years, which roughly matches the age range of the study sample. The current study used 10.3% as the comparator, which was the percentage of this subgroup with PHQ-9 scores of 10 or higher.
- *Incharge financial distress financial well-being (IFDFW).* A 2004 mail survey of the general US adult population (*N* = 1300) provided data for initial norms for the this scale. ^29^ The current study used the mean for the total sample (5.7) as the comparator.

*PTSD checklist for DSM-5 (PCL-5) with criterion A.* A previous study ^32^ administered the PCL-5 to 1822 infantry soldiers. The mean PCL-5 score was 11.8; 12.3% reached criterion for PTSD (PCL-5 score of 33 or higher), which was the norm used in the current study.

#### Substance use

One recent study found that 29.2% of 13,140 US veterans reported current tobacco use (*within last 30 days*) in the 2015 National Survey on Drug Use and Health. ^37^ The current study used 52.7% as the comparator for tobacco use, which was the rate for the veteran subgroup aged 26–34.

A recent survey asked 80 veterans who had been discharged from the service within the previous 12-month period about their current substance use (*over the past 6 months*). ^38^ The current study used the reported use rates of 88.6% for alcohol and 11.4% for illegal drugs as comparators.

Another study examined survey responses for 2587 veterans completing the 2014 National Survey on Drug Use and Health. ^39^ The comparator used in the current analysis was 13.6%, which was the weighted percentage for the past year cannabis use for veterans in the age group of 26–34 years.

The annual percentage of adults receiving an opioid prescription has been declining in the USA, both in the veteran population ^40^ and the general population. ^41^ The annual rate of opioid prescriptions was 16.1% for veterans in 2016 and 19.2% for the general population in 2018. The most recently published rate was used as the norm to compare with the self-reported use in the current sample.

### Statistical analyses

Exploratory data analyses were conducted on all relevant measures to determine their distributional properties, ^42^ examining the internal reliability (Cronbach’s alpha) for all standardized scales. Next, the analyses compared the study sample with population norms using *t* tests for continuous measures and chi-square tests for dichotomous measures. Effect sizes (*d*) for differences between the study sample mean ratings and published norms were calculated, using the standard formula for continuous measures ^43^ and the arcsine transformation for dichotomous measures. ^44^

Other analyses included a series of *t* tests and one-way analysis of variance tests to assess statistical associations of six demographic measures (sex, age, marital status, education, ethnicity, and race) and three measures related to military service (active duty status, combat duty service, disability rating) with the outcome measures. The analyses assessed associations by dichotomizing age (under 30, 30 and over), race (white, nonwhite), ethnicity (Hispanic, not Hispanic), and education (some college or less, college degree), and creating three subgroups for disability rating (0–60%, 70–80%, 90–100%). Univariate tests of significance with *p* values of .05 were followed by Bonferroni corrections.

## Results

### Sample characteristics

The sample consisted of 229 participants interviewed between May 2018 and June 2019. As shown in Table 1, 80% were male, 55% were under the age of 30, 46% were White, and 57% were married or cohabiting with a partner. Most respondents had completed high school but had not earned a college degree. Respondents lived in all regions of the USA, including 63% in the Southern region. Nearly 74% of the sample were recently discharged veterans (within the last year); the remainder were still on active duty with an estimated date of discharge within 6 months. Most respondents served in the army, although the other four branches were represented. About half (47%) served in a combat zone. (See Appendix 1. Table 4 in offline appendix for details of military service.)

**Table 1:** Background characteristics (*N* = 229)

Characteristic		*N* (%)
Sex	Male	184 (80.3%)
	Female	44 (19.2%)
	Other	1 (0.4%)
Age	<30 years old	126 (55.0%)
	≥30 years old	103 (45.0%)
Marital status	Married/cohabiting partner	131 (57.2%)
	Divorced/separated	39 (17.0%)
	Never married	59 (25.8%)
Race	White	106 (46.3%)
	Black or African American	83 (36.2%)
	Asian	15 (6.6%)
	American Indian or Alaskan Native	2 (0.9%)
	Hawaiian/Pacific Islander	4 (1.8%)
	Other	34 (14.9%)
Ethnicity	Hispanic, Latino, or Spanish	44 (19.2%)
Education	High school diploma/GED	34 (14.8%)
	Technical certificate/some college	113 (49.3%)
	Associate degree	29 (12.7%)
	Bachelor’s degree	32 (14.0%)
	Masters, PhD, or professional degree	21 (9.2%)
Current residence	House/apartment with someone	149 (65.1%)
	House/apartment living alone	23 (10.0%)
	Living with family	34 (14.8%)
	Staying with friends/transient	7 (3.1%)
	Military housing	11 (4.8%)
	Other	5 (2.2%)

Overall, 163 participants reported disability ratings at baseline or in a follow-up interview (71% of the total sample). The mean disability rating for the sample was 73% (*SD* = 22.6). The sample included 58 (36%) with a disability rating of 90% or 100%; 58 (36%) with a rating of 70% or 80%; and 47 (29%) with a rating between 0 and 60%. In the sample reporting disabilities ratings, 161 indicated the type of disability. A total of 102 (63%) respondents indicated they had disability ratings for both mental and physical reasons; 6 (4%) reported mental disability ratings only, and 55 (34%) reported physical disability ratings only.

### Comparison of health and well-being of study sample with published norms

#### Health and well-being

Table 2 shows comparisons of mean ratings for the study sample to population norms. According to the SWLS scale anchors, the study sample was on average “slightly satisfied with life,” reporting significantly less satisfaction compared to a sample of US veterans and service members. ^35^ The effect size for the difference in mean satisfaction score was large.

**Table 2:** Health and well-being: comparisons between study sample (*n*=229) and population norms

Measure/scale	Study sample *M* (*SD*)	Population norm *M* (*SD*)	Test of significance and *d* effect size	Citation	Population description
SWLS ^**a**^	22.3 (7.2)	28.0 (6.1)	*t* = 7.72, *p* < .001 *d* = .85	Robertson & Brott (2014)	US military in transition (*N* =136)
VR-12 mental component (MCS) ^**b**^	42.9 (14.6)	50.1 (11.5)	*t* = 9.37, *p* < .001 *d* = .55	Selim et al. (2009)	National US (*N* = 173,221)
VR-12 physical component (PCS) ^**b**^	40.7 (10.8)	39.8 (12.3)	*t* = 1.08, *p* = .28	Selim et al. (2009)	National US (*N* = 173,221)
PHQ-9 ^c^ % screen positive for moderate depression (score ≥10)	41.9%	10.3%	*χ*^2^=54.73, p < .001 *d* = .74	Liu et al. (2019)	U.S. veterans aged 25 to 44 (*N* =204)
IFDFW ^d^	5.32 (2.60)	5.70 (2.40)	*t* = 2.18, *p* = .03 *d* = .15	Garman et al. (2005)	National US (*N* =1300)
PCL-5 ^**e**^ % screen positive for PTSD (Score ≥ 33)	28.2 (20.9) 49/163 30.1%	11.8 (16.0) 216/1751 12.3%	*t* = 14.07, *p* < .001 *d* = .88 χ^2^=39.28, *p* <.001 *d* = .44	Hoge et al. (2014)	US infantry soldiers (*N* = 1822)

^**a**^Satisfaction with life scale: scores range from 1 (low satisfaction) to 7 (high satisfaction)

^**b**^Higher scores indicate better health. *N* = 163 in age group 25–34; *N* = 66 in age group 35–44

^**c**^Patient health questionnaire-9: ≤4: minimal; 5–9: mild; 10–14: moderate; 15–19: moderately severe; ≥20: severe depression

^**d**^Incharge financial distress/financial well-being scale: 1.0 = high distress/low well-being – 10.0 = low distress/high well-being

^e^PTSD Checklist for DSM-5 with criterion A

Based on the MCS, the study sample reported significantly poorer mental health than the population norm. The effect size for this difference was moderate. The mean study sample rating on physical health (the PCS) did not differ from the published norm.

Compared to a sample of US veterans, the percentage of respondents with moderate depression, as measured by the PHQ-9, was four times greater (42% versus 10%). ^36^ The study sample included 42 (18.3%) respondents who reported severe or moderately severe depression.

The study sample also had significantly more financial distress than a national sample, ^45^ though the effect size was small. The study sample was predominantly male; the IFDFW norm for men is substantially higher than for the general population (6.2 versus 5.7); restricting the comparison to men increased the effect size in financial distress between the study sample and the comparator (*d* = .30).

Finally, respondents reported significantly greater symptoms of PTSD than a comparison group of US infantry soldiers. ^32^ Similarly, the percentage of respondents scoring above the PCL-5 criterion score for PTSD was significantly higher for the study group compared to the published norm.

During the last year, alcohol was the most widely consumed of all substances in the study sample, with 81% drinking on at least one occasion, followed by prescribed opioids (42%), tobacco (38%), marijuana (14%), and illegal drug use (2%), as shown in Table 3. As shown in Appendix 2. Table 5 (see offline appendix), one-fifth of the sample reported having 6 or more alcoholic drinks in one day during the last month. Prescription opioid use was very high (42%) in the study sample, more than twice the rate of 19% of the general adult population who were prescribed opioids during 2018. Moreover, 14% reported using prescription opioids daily for 3 months or more in the last year. Aside from opioid use, patterns of substance use for the study sample did not differ substantively from published norms.

**Table 3:** Comparisons between study sample and published findings on substance use

Substance	Total (*n*=229), *n* (%)	Population norm, *n* (%)	Test of significance	Citation	Population description
Tobacco	86 (37.6%)	52.7%	*χ*^2^=21.07, *p* < .001 (one-sample test)	Odani et al. (2018)	US veterans aged 26–34 (*N* not reported; subgroup from a sample of 13,140)
Alcohol	186 (81.2%)	88.6%	*χ*^2^=2.40, *p* = .12	Derefinko et al. (2018)	US veterans (*N* = 80)
Marijuana and cannabis products	32 (14.0%)	13.6%	*χ*^2^= 0.02, *p* = .88	Davis et al. (2018)	US veterans (*N* = 2587)
Illegal drugs (cocaine, meth, opiates, etc.)	5 (2.2%)	11.4%	*χ*^2^=11.27, *p* < .001	Derefinko et al. (2018)	US veterans (*N* =80)
Prescribed opioids	96 (41.9%)	19.2%	*χ*^2^=76.07, *p* < .001 (one-sample test)	Lin et al. (2020)	National retail pharmaceutical database

In summary, the study sample reported significantly poorer outcomes than population-based norms on 5 of 6 standardized measures of health and well-being. The strongest findings were for self-reported depression and PTSD. On both of these measures, the study sample reflected high symptom severity and very high rates of scoring above screening thresholds.

### Background characteristics as correlates of health, well-being, and substance use

As shown in Appendix 3. Table 6 (see offline appendix), a series of exploratory analyses were conducted to identify potentially significant correlates of health and well-being. Most comparisons did not reach statistical significance even at *p* < .05 and only one reached significance after Bonferroni correction.

## Discussion

This study examined a sample of enlisted men and women with high levels of service-connected disabilities who either had recently transitioned or were soon to transition from the military to civilian life and who had voluntarily enrolled in a national evaluation of an innovative program to help veterans find employment. The ethnically and racially diverse sample represented all branches of the US military. Most study participants had high cumulative disability ratings, consistent with trends in recent statistics for post 9/11 veterans. ^19^ Study participants resided throughout the USA with a majority concentrated in the South, consistent with national statistics for veterans (https://www.va.gov/vetdata/docs/Maps/VetPop16_PopStateFY19.pdf). The current study described the extent of mental and physical health conditions, financial insecurity, and substance use in this sample of respondents with disabilities. Lacking a matched comparison group of respondents without disabilities, study findings were compared with available published norms based on surveys of the general civilian population, active duty soldiers, or veterans. For each measure, the single most comprehensive study defining norms in the study population was identified.

Comparisons on six standardized self-report measures documented high levels of distress in the study sample. Many respondents were moderately or severely depressed and reported PTSD symptoms. Respondents also reported financial distress and overall dissatisfaction with life. The only standardized scale on which the study sample did not differ from published norms was self-reported physical health limitations, even though 97% of the sample reported physical reasons for some or all their disability ratings.

Consistent with reported physical disabilities, prescribed opioid use was twice the opioid prescription rate in the general population. Nearly one-third of the 96 study participants prescribed opioid medications reported daily use for 3 months or more in the past year. The obvious concerns with prescribed opioid use are addiction and overdose. Opioid addiction often starts with prescribed medications; for example, two-thirds of heroin users initially used prescription opioids. ^41^ In 2017, 36% of opioid overdose deaths in the USA involved prescription drugs. ^41^ The VA has taken steps to reduce prescription of opioids, decreasing the number of veterans prescribed opioids by 64%, from 679,000 in 2012 to 247,000 in 2020. ^46^

Aside from opioid use, reported substance use was similar in the study sample to published norms. However, 80% of participants reported drinking alcohol, including 20% of the sample reported having 6 or more alcoholic drinks in a single day in the last month. Self-reported substance use varies greatly from study to study, undoubtedly influenced by the context in which the interview is administered. For example, active duty respondents are unlikely to report illegal substance use for obvious reasons.

Age, sex, race, and marital status were not associated with measures of health and well-being. In addition, neither serving in a combat zone nor cumulative disability ratings was associated with health outcomes. Prior research has been mixed regarding whether combat duty in itself adversely affects mental health and well-being. ^47,48^

During and after discharge from military service, many veterans with service-connected disabilities experience significant psychological distress. ^17^ This study found that levels of depression, PTSD symptoms, and financial distress were significantly higher than found in published norms, even in the active duty subsample, although the differences were greater in the post-discharge veteran subsample. These findings suggest that mental health problems may be more likely to emerge or become more serious *after* discharge from the military.

The study findings highlight the need for access to appropriate evidence-based mental health treatment as well as transition services to help manage the challenges of returning to civilian life. Veterans who have recently transitioned to civilian life, especially those with depression, PTSD, and financial stress, may be at risk for poorer functioning. ^47^ Poorly managed transitions create critical risks for veterans, including suicide, homelessness, and worsening physical and mental health. Continuous improvement of transition services should be a major public policy goal. Furthermore, the foregone tax revenue that occurs when veterans transition poorly are unknown but likely large.

### Study limitations

This study had two main limitations: (1) the sampling method resulted in a self-selected sample that was not representative of the population of transitioning veterans, and (2) the study lacked a single comparison sample matched on appropriate background characteristics. The study examined an opportunity sample of enlisted men and women during the transition period from military service who volunteered for a study promising to help them obtain competitive employment. As a self-selected sample of relatively young participants who enrolled in a project offering vocational assistance, the findings cannot be generalized to the population of service members transitioning out of the military. Furthermore, because unemployment has been associated with poorer mental health outcomes, the findings might be explained by the fact that the sample consisted of unemployed (or marginally employed) participants. ^49^

The study sample was compared with published findings of a variety of surveys, each with its own sampling methods. The statistical comparisons with published norms did not control for demographic or other possible confounding factors, so these comparisons should be viewed with caution. However, this concern may be partially allayed by the fact that background factors were generally not associated with scale ratings or substance use in the study sample.

## Implications for Behavioral Health

Among the 200,000 US service members discharged annually are a sizeable proportion with serious behavioral health symptoms and financial needs, contributing to alarming rates of addiction, isolation, suicide, homelessness, and other adverse outcomes. It is recommended that federal and state policymakers facilitate access to a range of evidence-based services, including behavioral health treatment, employment programs, and perhaps nonmedical interventions such as peer supports to address the transition to civilian life. The VA healthcare system has an important role in addressing these needs, but a substantial percentage (39%) of veterans never access VA health care, ^50^ and roughly one-third of veterans who do initially enroll in VA healthcare drop out without receiving treatment after receiving a psychiatric diagnosis. ^51^ Veterans also access numerous nongovernmental resources. For example, one survey identified over 20,000 public and private programs available to veterans that provide help for legal, housing, financial, health care, social connectedness, and other needs.^3^ These alternatives appeal to many veterans but are fragmented like much of the health care and social service systems in the USA. Further research is needed to determine which programs and services are effective and for which veteran subgroups. Not everyone is likely to benefit from the same service options. Some may benefit primarily from employment services, others from mental health services, others may prefer and receive help through self-help and online services, and some veterans may benefit from a combination of these.

## Appendix 1

**Table 4:** Military experience

Measure		*N* (%)
Active duty status	Active duty	60 (26.2%)
	Recently discharged	169 (73.8%)
Military branch	Army	144 (62.9%)
	Air Force	31 (13.5%)
	Navy	30 (13.1%)
	Marine Corp	20 (8.7%)
	Coast Guard	4 (1.7%)
Rank at discharge	E1-E3	23 (10.0%)
	E4	84 (36.7%)
	E5	53 (23.1%)
	E6-E8	69 (30.1%)
Age when first enlisted, *M* (*SD*)		21.5 (4.0)
Years served, *M* (*SD*)		8.6 (6.6)
Service included pre-9/11 period (pre-2001)		42 (18.3%)
Military service without interruption		215 (93.9%)
Served in a combat zone		108 (47.2%)

## Appendix 2

**Table 5:** Substance use in the study sample (*N* =229)

Tobacco	
Use of tobacco in the past year, *n* (%)	86 (37.6%)
Use per week (*N* = 86 users), *M* (*SD*)	43.0 (52.1)
Alcohol	
Consumption of alcohol in the past year, *n* (%)	186 (81.2%)
Six or more drinks in one day in the past month, *n* (%)	45 (19.7%)
Alcoholic drinks per week (*N* = 186 users), *M* (*SD*)	6.68 (9.27)
Marijuana and cannabis products	
Use of marijuana or cannabis in past year, *n* (%)	32 (14.0%)
Use per week (*N* = 32 users), *M* (*SD*)	17.7 (34.2)
Illegal drugs (cocaine, methamphetamines, opiates, etc.)	
Use of illegal drugs in past year, *n* (%)	5 (2.2%)
Use per week (*N* = 5 users), *M* (*SD*)	9.5 (6.4)
Opioids prescribed by a medical provider in the past year	
Prescribed opioid use in past year	96 (41.9%)
Duration of use (total sample)	
Never	133 (58.1%)
Monthly or less	33 (14.4%)
Daily for a week	18 (7.9%)
Daily for a month	14 (6.1%)
Daily for 3 months or more	31 (13.5%)

## Appendix 3

**Table 6:** Demographic and military service correlates of outcome

Outcome measure	Sex			Age			Marital status			
	Male	Female	Test of signif	<30	≥30	Test of signif	Married		Not married	Test of signif
	(*N*=184) *M* (*SD*)/*N* (%)	(*N*=44) *M* (*SD*)/*N* (%)		(*N*=126) *M* (SD)/*N* (%)	(*N*=103) *M* (*SD*)/*N* (%)		(*N*=131) *M* (*SD*)/*N* (%)		(*N*=98) *M* (*SD*)/*N* (%)	
SWLS	22.53 (7.17)	21.66 (6.97)	*t*=0.73	22.33 (7.58)	22.29 (6.61)	*t*=0.036	23.24 (6.54)		21.06 (7.75)	t=2.31*
PCS (VR-12)	39.97 (10.96)	43.59 (10.02)	*t*=−2.00*	41.38 (10.85)	39.86 (10.83)	*t*=1.05	39.12 (10.93)		42.80 (10.41)	*t*=−2.57*
MCS (VR-12)	43.28 (14.91)	41.82 (13.33)	*t*=0.60	42.21 (14.48)	43.83 (14.78)	*t*=-0.836	44.12 (14.44)		41.36 (14.75)	*t*=1.42
PHQ-9 score	8.67 (6.38)	8.59 (5.58)	*t*=0.074	9.13 (6.28)	8.21 (6.27)	*t*=1.10	8.37 (6.28)		9.18 (6.27)	*t*=-0.98
PHQ-9: mod depressed	77 (41.8%)	18 (40.9%)	*χ* ^2^=0.013	57 (45.2%)	39 (37.9%)	*χ* ^2^=1.27	53 (40.5%)		43 (43.9%)	*χ* ^2^=0.27
IFDFW	5.45 (2.61)	4.71 (2.32)	*t*=1.72	5.18 (2.54)	5.49 (2.60)	*t*=−0.92	5.43 (2.51)		5.17 (2.65)	*t*=0.75
PCL-5 score	27.77 (21.26)	29.19 (19.16)	*t*=−0.342	29.42 (20.11)	26.86 (21.68)	*t*=0.79	27.08 (21.57)		29.82 (19.81)	*t*=−0.83
PCL-5 screen for PTSD	56 (41.8%)	12 (38.7%)	*χ* ^2^=0.099	38 (44.2%)	31 (38.8%)	*χ* ^2^=0.50	37 (37.4%)		32 (47.8%)	*χ* ^2^=1.78
Tobacco	74 (40.2%)	12 (27.3%)	*χ* ^2^=2.53	45 (35.7%)	41 (39.8%)	*χ* ^2^=0.41	47 (35.9%)		39 (39.8%)	*χ* ^2^=0.37
Alcohol	151 (82.1%)	34 (77.3%)	*χ* ^2^=0.53	101 (80.2%)	85 (82.5%)	*χ* ^2^=0.21	105 (80.2%)		81 (82.7%)	*χ* ^2^=0.23
Cannabis products	28 (15.2%)	3 (6.8%)	*χ* ^2^=2.41	21 (16.7%)	11 (10.7%)	*χ* ^2^=2.84	11 (8.4%)		21 (21.4%)	χ^2^=9.44**
Illegal drugs	3 (1.6%)	1 (2.3%)	*χ* ^2^=0.32	3 (2.4%)	2 (1.9%)	*χ* ^2^=1.28	1 (0.8%)		4 (4.1%)	*χ* ^2^=4.27
Prescribed opioids	78 (42.4%)	17 (38.6%)	*χ* ^2^=0.21	45 (35.7%)	51 (49.5%)	*χ* ^2^=4.43*	57 (43.5%)		39 (39.8%)	*χ* ^2^=0.32
Outcome measure	Race			Ethnicity			Education			
	White	Nonwhite	Test of signif	Hispanic	Not Hispanic	Test of signif	High school or some college		College degree	Test of signif
	(*N*=106) *M* (*SD*)/*N* (%)	(*N*=123) *M* (*SD*)/*N* (%)		(*N*=44) *M* (*SD*)/*N* (%)	(*N*=184) *M* (*SD*)/*N* (%)		(*N*=147) *M* (*SD*)/*N* (%)		(*N*=82) *M* (*SD*)/*N* (%)	
SWLS	22.70 (6.84)	21.98 (7.41)	*t*=0.76	21.64 (7.75)	22.54 (6.96)	*t*=−.76	21.58 (7.60)		23.62 (6.08)	*t*=−2.23*
PCS (VR-12)	39.39 (11.15)	41.82 (10.49)	*t*=−1.70	41.31 (11.65)	40.64 (10.63)	*t*=.37	40.30 (10.70)		41.41 (11.13)	*t*=−0.74
MCS (VR-12)	41.50 (15.22)	44.17 (14.00)	*t*=−1.38	38.98 (15.03)	44.02 (14.29)	*t*=−2.08*	40.75 (15.21)		46.86 (12.61)	*t*=−3.26**
PHQ-9 score	9.79 (6.56)	7.79 (5.89)	*t*=2.44*	10.59 (6.97)	8.20 (5.97)	*t*=2.31	9.74 (6.16)		6.88 (6.10)	*t*=3.38**
PHQ-9: mod depressed	53 (50.0%)	43 (35.0%)	*χ* ^2^=5.29*	23 (52.3%)	72 (39.1%)	*χ* ^2^=2.52	71 (48.3%)		25 (30.5%)	*χ* ^2^=6.86**
IFDFW	5.56 (2.56)	5.11 (2.57)	*t*=1.33	5.35 (2.36)	5.33 (2.62)	*t*=0.06	4.70 (2.47)		5.94 (2.63)	*t*=−2.78**
PCL-5 score	28.74 (21.41)	27.55 (20.32)	*t*=0.37	33.41 (22.59)	26.84 (20.26)	*t*=1.65	32.06 (21.16)		21.86 (18.87)	*t*=3.14**
PCL-5 screen for PTSD	38 (42.7%)	31 (40.3%)	*χ* ^2^=0.10	18 (52.9%)	51 (38.6%)	*χ* ^2^=2.28	53 (51.5%)		16 (19.5%)	*χ* ^2^=10.93**
Tobacco	49 (46.2%)	37 (30.1%)	*χ* ^2^=6.33*	16 (36.4%)	70 (38.0%)	*χ* ^2^=0.04	60 (40.8%)		26 (31.7%)	*χ* ^2^=1.86
Alcohol	91 (85.8%)	95 (77.2%)	*χ* ^2^=2.77	30 (68.2%)	156 (84.8%)	*χ* ^2^=6.51*	117 (79.6%)		69 (84.1%)	*χ* ^2^=0.72
Cannabis products	17 (16.0%)	15 (12.2%)	*χ* ^2^=1.91	11 (25.0%)	21 (11.4%)	*χ* ^2^=5.62	22 (15.0%)		10 (12.2%)	*χ* ^2^=0.92
Illegal drugs	3 (2.8%)	2 (1.6%)	*χ* ^2^=1.57	1 (2.3%)	4 (2.2%)	*χ* ^2^=.24	2 (1.4%)		3 (3.7%)	*χ* ^2^=1.85
Prescribed opioids	38 (35.8%)	58 (47.2%)	*χ* ^2^=3.00	17 (38.6%)	78 (42.4%)	*χ* ^2^=.21	57 (38.8%)		39 (47.6%)	*χ* ^2^=1.67
Outcome measure	Active duty status	Veteran	Test of signif	Yes	No	Test of signif	0–60%	70-80%	90–100%	Test of signif
	(*N*=60) *M* (*SD*)/*N* (%)	(*N*=169) *M* (*SD*)/*N* (%)		*N*=108) *M* (*SD*)/*N* (%)	(N=120) *M* (*SD*)/*N* (%)		(*N*=47) *M* (*SD*)/*N* (%)	(N=58) *M* (*SD*)/*N* (%)	*N*=58) *M* (*SD*)/*N* (%)	
SWLS	24.67 (5.91)	21.47 (7.38)	*t*=3.44**	22.35 (6.71)	22.18 (7.51)	*t*=0.18	22.81 (7.22)	22.86 (5.86)	22.36 (7.51)	*F*=0.090
PCS (VR-12)	41.75 (11.29)	40.31 (1.48)	*t*=0.88	40.19 (11.31)	41.07 (10.44)	*t*=−0.61	42.42 (10.17)	39.81 (11.23)	38.18 (10.99)	*F*=1.99
MCS (VR-12)	47.74 (14.04)	41.19 (14.45)	*t*=3.06**	42.58 (14.89)	43.10 (14.35)	*t*=−0.27	44.21 (14.13)	43.31 (13.95)	43.34 (13.91)	*F*=0.068
PHQ-9 score	6.44 (5.69)	9.54 (6.29)	*t*=−3.38**	8.98 (6.39)	8.53 (6.19)	*t*=0.54	8.15 (6.52)	8.29 (6.17)	9.53 (6.39)	*F*=0.80
PHQ-9: mod depressed	16 (26.2%)	80 (47.6%)	*χ* ^2^=8.41**	46 (42.6%)	50 (41.7%)	χ^2^=0.75	19 (40.4%)	21 (36.2%)	27 (46.6%)	*χ* ^2^=1.30
IFDFW	6.90 (2.01)	4.74 (2.51)	*t*=6.68***	5.56 (2.51)	5.08 (2.61)	*t*=1.40	5.07 (2.73)	5.76 (2.29)	5.44 (2.72)	*F*=0.95
PCL-5 score	21.02 (20.85)	30.85 (20.31)	*t*=−2.75**	30.93 (21.45)	25.68 (19.91)	*t*=1.63	23.27 (19.73)	29.00 (21.29)	28.22 (20.66)	*F*=0.87
PCL-5 screen for PTSD	12 (26.7%)	57 (47.1%)	*χ* ^2^=5.64*	40 (47.6%)	29 (35.8%)	*χ* ^2^=3.09	10 (27.0%)	16 (41.0%)	21 (46.7%)	*χ* ^2^=3.41
Tobacco	21 (34.4%)	65 (38.7%)	*χ* ^2^=0.35	44 (40.7%)	41 (34.2%)	*χ* ^2^=2.72	17 (36.2%)	18 (31.0%)	23 (39.7%)	*χ* ^2^=0.95
Alcohol	47 (77.0%)	139 (82.7%)	*χ* ^2^=0.95	91 (84.3%)	94 (78.3%)	*χ* ^2^=1.54	36 (76.6%)	46 (79.3%)	47 (81.0%)	*χ* ^2^=0.31
Cannabis products	0 (0.0%)	32 (19.0%)	*χ* ^2^=14.00**	15 (13.9%)	17 (14.2%)	*χ* ^2^=1.29	8 (17.0%)	10 (17.2%)	8 (13.8%)	*χ* ^2^=2.17
Illegal drugs	0 (0.0%)	5 (3.0%)	*χ* ^2^=2.24	2 (1.9%)	3 (2.5%)	*χ* ^2^=1.25	0 (0.0%)	2 (3.4%)	1 (1.7%)	*χ* ^2^=3.57
Prescribed opioids	29 (47.5%)	67 (39.9%)	*χ* ^2^=1.08	53 (49.1%)	43 (35.8%)	*χ* ^2^=4.82	13 (27.7%)	25 (43.1%)	31 (53.4%)	*χ* ^2^=7.10*

^*^ *p* < .05, ^**^*p* < .01, ^***^*p* < .001

## References

[1] Government Accounting Office (2019). *Transitioning Service Members: Information on Military Employment Assistance Centers*.

[2] Morin R (2011). *The Difficult Transition from Military to Civilian Life*.

[3] Perkins DF, Aronson KR, Morgan NR (2020). Veterans’ use of programs and services as they transition to civilian life: Baseline assessment for the Veteran Metrics Initiative. Journal of Social Service Research..

[4] Kintzle S, Rasheed JM, Castro CA (2016). *The State of the American Veteran: The Chicagoland Veterans Study*.

[5] Taylor P, Morin R, Parker K (2011). *War and Sacrifice in the Post-9/11 Era The Military-Civilian Gap*.

[6] Mobbs MC, Bonanno GA (2018). Beyond war and PTSD: The crucial role of transition stress in the lives of military veterans. Clinical Psychology Review..

[7] Castro CA, Kintzle S, Hassan A (2013). *The State of the American Veteran: The Los Angeles County Veterans*.

[8] Castro CA, Kintzle S, Hassan A (2015). *The State of the American Veteran: The Orange County Veterans Study*.

[9] Castro CA, Kintzle S (2017). *The State of the American Veteran: The San Francisco Veterans Study*.

[10] Hamilton AB, Williams L, Washington DL (2015). Military and mental health correlates of unemployment in a national sample of women veterans. Medical Care..

[11] Zivin K, Campbell DG, Lanto AB (2012). Relationships between mood and employment over time among depressed VA primary care patients. General Hospital Psychiatry..

[12] Burnett-Zeigler I, Ilgen MA, Bohnert K (2013). The impact of psychiatric disorders on employment: Results from a national survey (NESARC). Community Mental Health Journal..

[13] Blore JD, Sim MR, Forbes AB (2015). Depression in Gulf War veterans: A systematic review and meta-analysis. Psychological Medicine..

[14] Chandrasekaran R. A legacy of pride and pain. *The **Washington Post.* March 29, 2014. Available online at http://www.washingtonpost.com/sf/national/2014/03/29/a-legacy-of-pride-and-pain/. Accessed 1 Oct 2021.

[15] McNally RJ, Frueh BC (2013). Why are Iraq and Afghanistan War veterans seeking PTSD disability compensation at unprecedented rates?. Journal of Anxiety Disorders..

[16] Oster C, Morello A, Venning A (2017). The health and wellbeing needs of veterans: A rapid review. BMC Psychiatry..

[17] Vogt DS, Tyrell FA, Bramande EA (2020). U.S. military veterans’ health and well-being in the first year after service. American Journal of Preventive Medicine.

[18] Lin LA, Peltzman T, McCarthy JF (2019). Changing trends in opioid overdose deaths and prescription opioid receipt among veterans. American Journal of Preventive Medicine..

[19] U.S. Bureau of Labor Statistics. *Employment Situation of Veterans—2019.* Publication No. USDL-20-0452. Washington: U.S. Department of Labor, U.S. Government Printing Office; 2020.

[20] Tsai J, Rosenheck RA (2016). US veterans’ use of VA mental health services and disability compensation increased from 2001 to 2010. Health Affairs..

[21] Prokos A, Cabage LN (2015). Women military veterans, disability, and employment. Armed Forces and Society..

[22] Davis LL, Kyriakides TC, Suris A (2018). Veterans individual placement and support towards advancing recovery: Methods and baseline clinical characteristics of a multisite study. Psychiatric Rehabilitation Journal..

[23] Diener E, Emmons RA, Larsen RJ (1985). The Satisfaction With Life Scale. Journal of Personality Assessment.

[24] Pavot W, Diener E (1993). Review of the Satisfaction With Life Scale. Psychological Assessment..

[25] Selim AJ, Rogers W, Fleishman JA (2009). Updated U.S. population standard for the Veterans RAND 12-item Health Survey (VR-12). Quality of Life Research.

[26] Salyers MP, Bosworth HB, Swanson JW (2000). Reliability and validity of the SF-12 health survey among people with severe mental illness. Medical Care..

[27] Ware JE, Kosinkski M, Keller S (1996). A 12-item short-form health survey: Construction of scales and preliminary tests of reliability and validity. Medical Care..

[28] Kroenke K, Spitzer RL (2002). The PHQ-9: A new depression diagnostic and severity measure. Psychiatric Annals..

[29] Prawitz AD, Garman ET, Sorhaindo B (2006). In Charge Financial Distress/Financial Well-being Scale: Development, administration, and score interpretation. Journal of Financial Counseling and Planning..

[30] Garman ET, MacDicken B, Hunt H (2007). Progress in measuring changes in financial distress and financial well-being as a result of financial literacy programs. Consumer Interests Annual..

[31] Bovin MJ, Marx BP, Weathers FW (2016). Psychometric properties of the PTSD Checklist for Diagnostic and Statistical Manual of Mental Disorders–Fifth Edition (PCL-5) in veterans. Psychological Assessment..

[32] Hoge CW, Riviere LA, Wilk JE (2014). The prevalence of post-traumatic stress disorder (PTSD) in US combat soldiers: A head-to-head comparison of DSM-5 versus DSM-IV-TR symptom criteria with the PTSD checklist. Lancet Psychiatry..

[33] Weathers FW, Litz BT, Keane TM, et al. The PTSD Checklist for DSM-5 (PCL-5) – Extended Criterion A [Measurement instrument]. National Center for PTSD. 2013. Available online at https://www.ptsd.va.gov/professional/assessment/adult-sr/ptsd-checklist.asp. Accessed 1 Oct 2021.

[34] Kobau R, Sniezek J, Zack MM (2010). Well-being assessment: An evaluation of well-being scales for public health and population estimates of well-being among US adults. Applied Psychology: Health and Well-Being..

[35] Robertson HC, Brott PE (2014). Military veterans’ midlife career transition and life satisfaction. Professional Counselor..

[36] Liu Y, Collins C, Wang K (2019). The prevalence and trend of depression among veterans in the United States. Journal of Affective Disorders..

[37] Odani S, Agaku IT, Graffunder CM (2018). Tobacco product use among military veterans — United States, 2010–2015. MMWR and Morbidity and Mortality Weekly Report..

[38] Derefinko KJ, Hallsell TA, Isaacs MB (2018). Substance use and psychological distress before and after the military to civilian transition. Military Medicine..

[39] Davis AK, Lin LA, Ilgen MA (2018). Recent cannabis use among Veterans in the United States: Results from a national sample. Addictive Behaviors..

[40] Hadlandsmyth K, Mosher H, Vander Weg MW (2018). Decline in prescription opioids attributable to decreases in long-term use: A retrospective study in the Veterans Health Administration 2010–2016. Journal of General Internal Medicine..

[41] Schieber LZ, Guy GP, Seth P (2020). Variation in adult outpatient opioid prescription dispensing by age and sex — United States, 2008–2018. Morbidity and Mortality Weekly Report..

[42] Tukey JW (1977). *Exploratory Data Analysis*.

[43] Cohen J (1988). *Statistical Power Analysis for the Behavioral Sciences*.

[44] Lipsey MW (1990). *Design Sensitivity*.

[45] Garman ET, Sorhaindo B, Prawitz AD (2005). Development of and norms for the InCharge Financial Distress/Financial Well-Being Scale: A summary. Financial Counseling and Planning..

[46] Department of Veterans Affairs (2020). *VA Reduces Prescription Opioid Use by 64% During Past Eight Years*.

[47] Fortenbaugh FC, Fonda JR, Fortier CB (2020). The impact of common psychiatric and behavioral comorbidities on functional disability across time and individuals in post-9/11 veterans. Journal of Traumatic Stress..

[48] Hoopsick RA, Homish DL, Collins RL (2020). Is deployment status the critical determinant of psychosocial problems among reserve/guard soldiers?. Psychological Services..

[49] Paul KI, Moser K (2009). Unemployment impairs mental health: Meta-analyses. Journal of Vocational Behavior..

[50] Derefinko KJ, Hallsell TA, Isaacs MB (2019). Perceived needs of veterans transitioning from the military to civilian life. Journal of Behavioral Health Services and Research..

[51] Seal KH, Maguen S, Cohen B (2010). VA mental health services utilization in Iraq and Afghanistan veterans in the first year of receiving new mental health diagnoses. Journal of Traumatic Stress..

